# Microarray-Based Transcriptional Profiling of Renieramycin M and Jorunnamycin C, Isolated from Thai Marine Organisms

**DOI:** 10.3390/md7040483

**Published:** 2009-10-19

**Authors:** Kornvika Charupant, Khanit Suwanborirux, Naomi Daikuhara, Masashi Yokoya, Rie Ushijima-Sugano, Takatoshi Kawai, Takashi Owa, Naoki Saito

**Affiliations:** 1 Department of Pharmacognocy and Pharmaceutical Botany, Center for Bioactive Natural Products from Marine Organisms and Endophytic Fungi (BNPME), Faculty of Pharmaceutical Sciences, Chulalongkorn University, Pathumwan, Bangkok 10330, Thailand; 2 Graduate School of Pharmaceutical Sciences, Meiji Pharmaceutical University, 2-522-1 Noshio, Kiyose, Tokyo 204-8588, Japan; 3 Tsukuba Research Laboratories, Eisai Co. Ltd., 5-1-3 Tokodai, Tsukuba, Ibaraki 300-2635, Japan

**Keywords:** renieramycin M, jorunnamycin C, marine sponge, oligonucleotide microarray, antitumor agent

## Abstract

Renieramycin M and jorunnamycin C, two isoquinolinequinone compounds differing only at the C-22 ester side chain, were evaluated for their cytotoxic effects on human colon (HCT116) and breast (MDA-MB-435) cancer cell lines. These two compounds displayed potent cancer cell growth inhibition, their IC_50_ values reaching nanomolar order. To examine their effects on transcription, we carried out oligonucleotide microarray analysis with focus on the similarities and differences between the two compounds in terms of transcriptional profiles. We found that the down-regulation of PTPRK (protein tyrosine phosphatase receptor type K) can be considered as a biomarker responsive to the cytotoxic effects of this class of antitumor marine natural products.

## 1. Introduction

**Figure 1.**
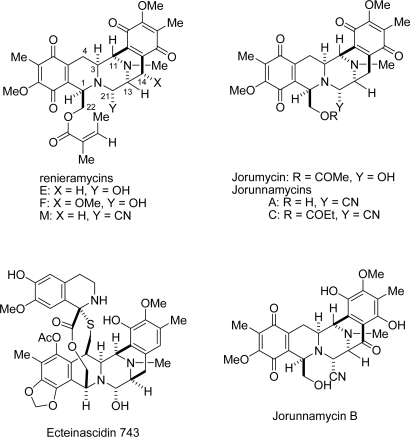
Structures of renieramycins, jorunnamycins, and related marine natural products with significant antiproliferative activity.

**Figure 2.**
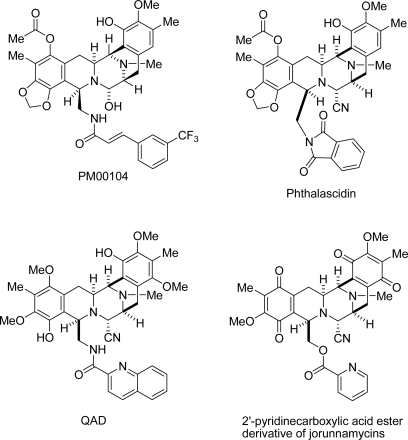
Structures of synthetic analogues maintaining significant high cytotoxicity.

Tetrahydroisoquinolinequinones and their reduced forms have attracted considerable interest over the past 30 years due to their potent biological activities [1]. A great many such natural products have been isolated, predominantly from Actinomycetes and marine organisms. On the basis of their characteristic structures, antibiotics could be subdivided into three categories: (1) naphthyridinomycins, cyanocyclines, and bioxalomycins, (2) saframycins and safracins, and (3) quinocarcins, tetrazomine, and lemonomycin. Meanwhile, marine natural products can be subdivided into two categories: (1) renieramycin, cribrostatin, jorumycin, and jorunnamycin and (2) ecteinascidins (Figure 1). The most bioactive member of marine natural isoquinoline family, ecteinascidin 743 (Yondelis, trabectedin), has a unique mechanism of action in that its binds to the minor groove of DNA to interfere with cell division, activated transcription, and DNA repair [2–9]. Ecteinascidin 743 has been approved by the European Commission for use in advanced soft tissue sarcoma patients who do not respond to anthracyclines and ifosfamide, or who are unsuited to receive these agents. The remarkable results of preclinical and clinical trials of ecteinascidin 743 have stimulated further research of this class of antitumor agents, including PM00104 (Zalypsis) [10,11], phthalascidin (Pt 650) [12], and QAD [13] (Figure 2).

Renieramycins E and F were isolated from *Reniera* sp. by Faulkner’s group in 1989 [14,15]. These two compounds, whose ring system and the relative stereochemistry are identical those of saframycin A, exhibit strong cytotoxicity toward cultured cells *in vitro* and antitumor activity against several experimental tumors *in vivo*. Jorumycin, which was discovered in very minute quantities from the mantle and mucus of the Pacific nudibranch *Jorunna funebris*, possesses growth inhibitory activity against various human cancer cell lines [16]. These three compounds have a relatively unstable carbinolamine group that may decomposed during isolation process, thus, they are available in only minute quantities from natural sources.

In our search for new metabolites via the isolation and characterization of biologically active compounds from Thai marine animals, we succeeded in the isolation and structure elucidation of renieramycin M, which is a stable congener of renieramycin E with α-aminonitrile group instead of calbinolamine group, from the Thai sponge, *Xestospongia* sp., by pretreatment with potassium cyanide [17,18]. We realized the gram-scale supply of renieramycin-type compounds using our procedure, and recently reported significant results gained from the extension of our initial investigation and the results of cytotoxicity evaluation of C-22 ester analogues and a very promising compound, the 2′-pyridinecarboxylic acid ester derivative [19] (Figure 2). We also reported the isolation of jorunnamycins A–C from the mantles, visceral organs, and egg ribbons of the Thai nudibranch *Jorunna funebris,* following the same procedure that used potassium cyanide [20].

Described herein is a significant extension of our initial investigation of renieramycin M and jorunnamycin C, cyano-group containing isoquinolinequinone ester analogues. We used oligonucleotide microarray analysis to clarify the effects of these two compounds on cellular transcription. We focused on transcriptional SAR (structure and activity relationship) studies to identify a potential gene expression marker(s) that is closely associated with the antitumor activity of these fascinating marine natural products.

## 2. Results and Discussion

Renieramycin M and jorunnamycin C were isolated from potassium cyanide pretreated sponge, *Xestospongia* sp., and nudibranch, *Jorunna funebris*, respectively. Human cancer cell lines HCT116 (colon) and MDA-MB-435 (breast) were grown in RMPI 1640 (Sigma) containing supplements that included 10% (v/v) heat-inactivated fetal bovine serum (Equitech-BIO) and a solution of 100 U/mL penicillin and 100 μg/mL streptomycin (Invitrogen). Cell culture was performed at 37 °C in a humidified atmosphere of 5% CO_2_ and 95% air.

After continuous exposure of these compounds for three days, the concentration required for 50% growth inhibition (IC_50_) was determined by the MTT colorimetric assay [21]. The results are presented in Table 1. In this assay, renieramycin M was more potent than jorunnamycin C (by approximately twofold on IC_50_ basis) against human HCT116 colon and MDA-MB-435 breast cancer cell lines [22].

In order to compare renieramycin M and jorunnamycin C on the basis of their transcriptional signatures, we analyzed the expression changes of more than 8,500 transcripts in HCT116 and MDA-MB-435 cells using Affymetrix Human Genome Focus arrays. The investigated time points were 4 h and 12 h. All data were obtained in triplicate to verify statistical significance [23].

**Figure 3.**
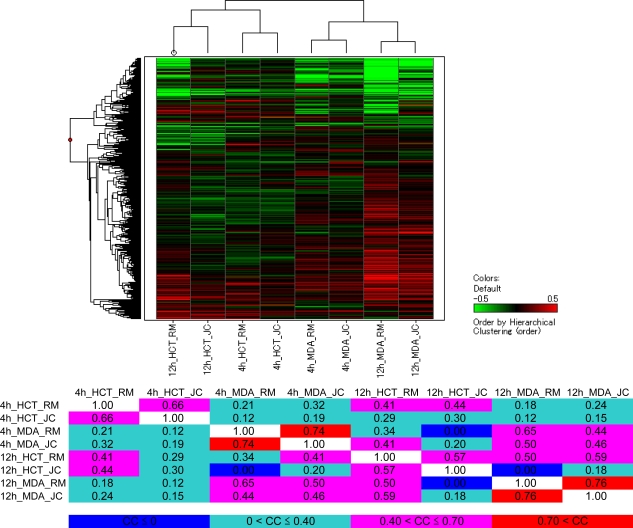
Correlation between transcriptional signatures of renieramycin M and jorunnamycin C. With respect to two human cancer cell lines (HCT116 and MDA-MB-435) and two time points (4 h and 12 h), hierarchical clustering data on the dendrogram format and cosine coefficient data on the table format are presented. Abbreviations: RM, renieramycin M; JC, jorunnamycin C; HCT, HCT116; MDA, MDA-MB-435; CC, correlation coefficient.

The hierarchical clustering data on the dendrogram format and the cosine coefficients between any two data points on the table format are shown in Figure 3. This analysis revealed that renieramycin M and jorunnamycin C have similar effects on the gene expression of each human cancer cell line and also at each time point (cosine coefficients: 0.66 in the 4-h treatment for HCT116; 0.57 in the 12-h treatment for HCT116; 0.74 in the 4-h treatment for MDA-MB-435; and 0.76 in the 12-h treatment for MDA-MB-435). The high correlation indicates that both compounds operate via essentially the same primary mechanism(s) of action, particularly in MDA-MB-435, a more sensitive cancer cell line to these antitumor agents.

**Figure 4.**
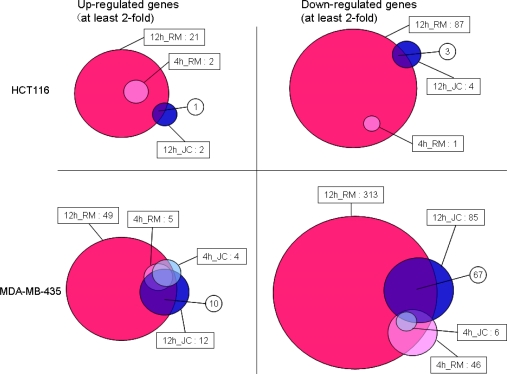
Venn diagrams showing the numbers of altered genes at least two fold by treatment with renieramycin M and jorunnamycin C in HCT116 and MDA-MB-435. Abbreviations: RM, renieramycin M; JC, jorunnamycin C.

Venn diagrams in Figure 4 present the number of genes up- and down-regulated by at least twofold with statistical significance (p-value < 0.05) by treatment with renieramycin M and jorunnamycin C. The obtained results are summarized as follows: (i) transcriptional down-regulation was more predominant than up-regulation for both compounds irrespective of the cell lines and the time points in general; (ii) the numbers of genes significantly altered by treatment with renieramycin M and jorunnamycin C were larger in MDA-MB-435 than in HCT116, consistent with the order of cellular sensitivity to both compounds; (iii) significant overlap was observed between the genes altered by treatment with renieramycin M and jorunnamycin C in both cell lines; and (iv) the numbers of significantly altered genes in both cell lines were larger with renieramycin M treatment than with jorunnamycin C treatment, even though the drug concentrations for this analysis were corrected by using the 2 × IC_50_ values of both compounds. These observations indicate that the C-22 ester side chain structure has a profound influence on the transcriptional perturbation by the renieramycin and jorunnamycin class of antitumor marine products.

**Figure 5.**
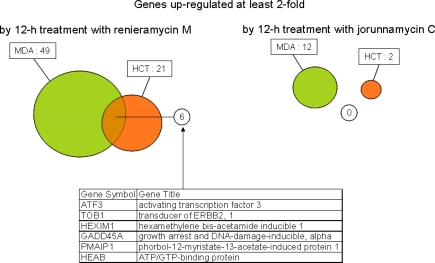
Genes up-regulated at least two fold coordinately in HCT116 and MDA-MB-435 by 12 h treatment with renieramycin M or jorunnamycin C. Abbreviations: HCT, HCT116; MDA, MDA-MB-435.

**Figure 6.**
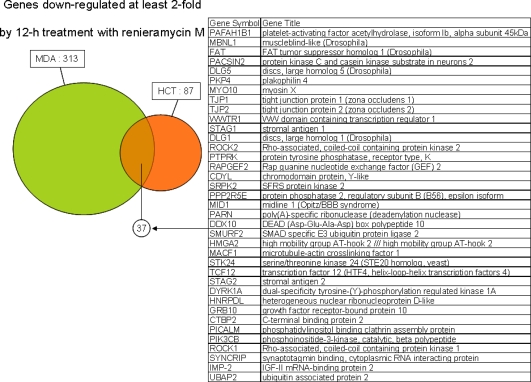

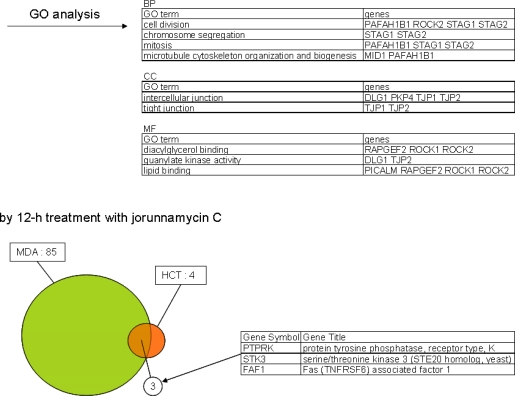
Genes down-regulated by at least two fold coordinately in HCT116 and MDA-MB-435 by 12 h treatment with renieramycin M or jorunnamycin C. Abbreviations: HCT, HCT116; MDA, MDA-MB-435; GO, gene ontology.

Figures 5 and 6 highlight the genes altered by at least twofold coordinately in HCT116 and MDA-MB-435 by 12-h treatment with renieramycin M or jorunnamycin C. The commonly up- or down-regulated genes between these two cell lines could be considered as biomarker candidates responsive to the cytotoxic effects of these compounds. In case of 12-h treatment with renieramycin M, there were 8 and 37 genes were found to be up- and down-regulated, respectively. It is note worthy that GADD45A was found to be up-regulated in three reports describing expression profiles of ecteinascidin 743 treatment cell lines, human epithelioid cervix adenocarcinoma HeLa [6], human lung carcinoma A549 [9], and HCT116 & MDA-MB-435 [12]. It is known that the transcription levels of GADD45A are increased following growth arrest and DNA-damage. This result strongly suggested that renieramycin M and jorunnamycin C have the similar G2/M arrest activities of ecteinascidin 743. On the other hand, down-regulated 37 genes of renieramycin M treatment experiments were subjected to GO (Gene Ontology) analysis to examine compound-associated biological processes, cellular components, and molecular functions. As the result, the following GO terms were enriched with p-values < 0.05: cell division, chromosome segregation, mitosis, and microtubule cytoskeleton organization and biogenesis in biological processes; intercellular junction and tight junction in cellular components; and diacylglycerol binding, guanylate kinase activity, and lipid binding in molecular functions (Figure 6). From this GO-biological processes analysis, it is suggested that renieramycin M may affect on mytotic phase of the cell cycle, which was also highlighted by the up-regulated GADD45A gene. GO analyses of the other set of expression level altered genes in common in both cell lines showed no significant enriched GO terms.

**Figure 7.**
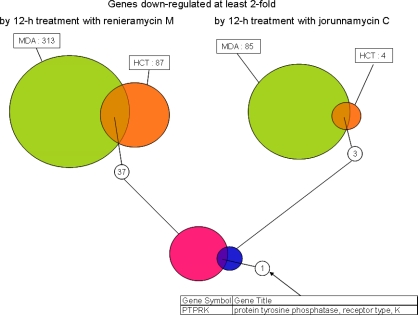
Identification of candidate biomarker genes down-regulated by at least two fold coordinately in HCT116 and MDA-MB-435 by 12 h treatments with renieramycin M and jorunnamycin C.

To select a set of biomarker genes in responsive to renieramycin M and jorunnamycin C treatments, the genes whose expression levels commonly altered in both cell lines with these two compounds were examined (Figure 7).

Within the list of down-regulated genes in 12-h treatments with both compounds, only one gene, *PTPRK*, was found to satisfy the selection criteria. The down-regulation of this gene seems to be involved in the primary mechanism(s) of action of both compounds, and is therefore considered to be a potential biomarker in response to the renieramycin and jorunnamycin class of antitumor marine products. *PTPRK* dephosphorylates *EGFR* and affects the downstream *Erk* activity. Xu *et al.* reported that overexpression of this gene in human keratinocytes decreased *EGFR* tyrosine phosphorylation, and resulted in near complete inhibition of growth [24]. This result is contrary to our expectation, however, the other PTPRs effects and kinase cascade cross-talks make the biological system to be complex, and the detailed *PTPRK* functions are still under the investigation. Martinez *et al.* reported that *PTPRK* is down-regulated in ecteinascidin 743 sensitive human sarcoma cells, on the contrary, it is up-regulated in resistant cells [25]. These findings support the idea that *PTPRK* gene is a potentially useful expression marker to monitor the antitumor effects of not only renieramycin M and jorunnamycin M but also a variety of naturally occurring isoquinolinequinones and their reduced forms with extremely potent antitumor activity.

## 3. Conclusions

We utilized oligonucleotide microarray analysis to profile the effects of renieramycin M and jorunnamycin C on cellular transcription, and found that the down-regulation of *PTPRK* gene can be a potentially useful biomarker in response to this class of unique antitumor marine products. The finding that renieramycin M is more potent against cancer cell growth than journnamycin C was also confirmed by the gene expression analysis, suggesting that the C-22 ester side chain structure should have a critical impact on not only the antiproliferative activity but also the transcriptional signatures of this class of unique antitumor natural products. This kind of microarray-based transcriptional SAR study represents a new and effective approach to drug discovery in the post-genomic era [26]. Effects to be examined of other types of cytotoxic molecules on the expression of *PTPRK* and compared with those of the ecteinascidin class of anticancer molecules are being made.

## Figures and Tables

**Table 1.** Antiproliferative activity of renieramycin M and jorunnamycin C against HCT116 and MDA-MB-435 human cancer cell lines.

**Compound**	**Human cancer cell line, IC_50_± SD (nM)**	
	**HCT116 (colon)**	**MDA-MB-435 (breast)**
Renieramycin M	16.4 ± 0.3	6.3 ± 0.1
Jorunnamycin C	27.3 ± 1.0	16.3 ± 1.3
